# Development and validation of a nomogram model for predicting clinical pregnancy in endometriosis patients undergoing fresh embryo transfer

**DOI:** 10.1186/s12884-023-06082-7

**Published:** 2023-10-31

**Authors:** Suqin Zhu, Xiuhua Liao, Wenwen Jiang, Yan Sun, Xiaojing Chen, Beihong Zheng

**Affiliations:** 1 Fujian Provincial Reproductive Medicine Center, Fujian Maternity and Child Health Hospital, College of Clinical Medicine for Obstetrics & Gynecology and Pediatrics, Fujian Medical University, No. 18 Daoshan Road, Fuzhou, Fujian Province 350001 China; 2 Fujian Maternal-Fetal Clinical Medicine Research Center, Fuzhou, China; 3 Fujian Key Laboratory of Prenatal Diagnosis and Birth Defect, Fuzhou, China

**Keywords:** Endometriosis, Predictive model, Clinical pregnancy, Nomogram, In vitro fertilization

## Abstract

**Purpose:**

To construct and validate a nomogram model for predicting clinical pregnancy in individuals with endometriosis undergoing fersh embryo transfer (ET).

**Methods:**

A retrospective analysis was conducted on 1630 individuals with endometriosis who underwent in vitro fertilization (IVF) with fresh embryo transfer at the Reproductive Medicine Center of Fujian Maternity and Child Health Hospital from January 2018 to January 2022. The research population was sorted into two groups through random sampling, namely, the model group (n = 1141) and the validation group (n = 489), with a ratio of 7:3. Univariate analysis was utilized to determine the influencing factors for clinical pregnancy in the model group. The LASSO algorithm was utilized to select the optimal matching factors, which were then included in a multifactorial forward stepwise logistic regression to determine independent influencing factors and develop a nomogram. The discrimination, accuracy, and clinical efficacy of the prediction model were analyzed utilizing the receiver operating characteristic (ROC) curve, calibration curve, and clinical decision curve.

**Results:**

Through multivariate-logistic-regression analysis, these factors were identified as independent influencing factors for the clinical pregnancy in endometriosis patients undergoing fresh embryo transfer: female age (OR = 0.933, 95% CI = 0.902–0.965, *P <* 0.001), ASRM stage (OR = 0.384, 95% CI = 0.276–0.532, *P < 0.001*), postoperative to IVF duration (OR = 0.496, 95% CI = 0.356–0.688, *P* < 0.001), antral follicle count (AFC) (OR = 1.076, 95% CI = 1.013–1.161, *P =* 0.045), anti-Müllerian hormone (AMH) (OR = 1.202, 95% CI = 1.073–1.35, *P =* 0.002), Gonadotrophin-releasing hormone (GnRH) agonist protocol (OR = 1.536, 95% CI = 1.109–2.131, *P* = 0.01), number of oocytes retrieved (OR = 1.154, 95% CI = 1.067–1.249, *P* < 0.001), number of high-quality cleavage embryos (OR = 1.261, 95% CI = 1.164–1.369, *P* < 0.001), and number of embryos transferred (OR = 1.957, 95% CI = 1.435–2.679, *P* < 0.001). A prediction model for estimating the clinical pregnancy probability in individuals with endometriosis was constructed per these identified independent factors. The ROC showed an area under the curve (AUC) of 0.807 (95% CI = 0.782–0.832) in the model group and 0.800 (95% CI = 0.761–0.84) in the validation group. The Hosmer-Lemeshow test demonstrated no statistically significant difference between predicted and actual clinical pregnancy probabilities (*P >* 0.05). The clinical decision curve demonstrated that both the model and the validation groups achieved maximum net benefit at threshold probability values of 0.08–0.96 and 0.16–0.96, indicating good clinical efficacy within this range of threshold probabilities.

**Conclusion:**

Female age, ASRM stage, postoperative to IVF duration, stimulation protocol, AFC, AMH, number of oocytes retrieved, number of high-quality cleavage embryos and number of transferred embryos are independent influencing factors for the clinical pregnancy rate in individuals with endometriosis receiving fresh embryo transfer. The nomogram model based on these factors demonstrates good clinical predictive value and efficacy, providing a basis for clinical prognosis, intervention, and individualized medical treatment planning.

## Introduction

Endometriosis is a common gynecological disorder that affects 10% of females in their reproductive age [1]. A characteristic pathological manifestation of endometriosis is the occurrence of endometrial tissue beyond the confines of the uterine cavity. Pelvic pain and infertility are the primary clinical manifestations of endometriosis. Around 40% of endometriosis patients experience infertility, while 70–85% of endometriosis patients suffer from pelvic pain. Furthermore, 25–48% of infertility patients and 71–87% of women with chronic pelvic pain have endometriosis [2]. endometriosis considerably impact the quality of life for affected individuals, leading to a notable reduction in their overall well-being. Additionally, it places a substantial economic burden on society.

Endometriosis is an important contributing factor to infertility [3]. A study involving 203 histologically confirmed cases of peritoneal endometriosis and 1,292 infertile patients without endometriosis (control group) found that the proportion of primary infertility was significantly elevated in the endometriosis group. However, the proportions of normal menstrual cycles and levels of AMH were similar between the two groups, suggesting that infertility in patients with mild endometriosis is unrelated to ovarian reserve function. This finding confirms that within the subgroup of infertile patients with regular menstrual cycles, patent fallopian tubes on at least one side, and normal routine semen analysis results, there exists a population without direct evidence of moderate to severe endometriosis. It is estimated that up to 50% of this subgroup may have mild endometriosis [4].

In clinical practice, accurately predicting the pregnancy outcomes of endometriosis patients undergoing IVF is crucial. Considering treatment impact, individual parameters, and laboratory tests, the development of an effective, convenient, and intuitive clinical predictive model enables healthcare professionals to estimate the probability of successful pregnancy based on specific patient conditions. This facilitates optimal treatment plan selection and personalized care while giving patients realistic expectations regarding their circumstances and the nomogram analysis results.

The current study successfully developed a predictive nomogram model by integrating independent influencing factors of pregnancy outcomes. The model quantifies, visualizes, and graphically represents the logistic regression results, enabling inference of variable values by graph and displaying continuous prediction probabilities [5]. It aims to obtain the probability of clinical pregnancy in individuals with endometriosis, providing improved guidance and facilitating clinical practice. For the above reasons, the present research retrospectively assessed the clinical and laboratory data of endometriosis patients throughout the entire IVF-ET process. Using the available data, a predictive nomogram model for clinical pregnancy in individuals with endometriosis was developed. The model was internally and clinically validated to ensure its accuracy and reliability. The main aim of this model is to guide prognosis and assist in the development of individualized treatment plans for endometriosis patients.

## Methodology

### Study population and grouping

A retrospective analysis was conducted on the clinical data of 1630 individuals with endometriosis who underwent fersh embryo transfer at the Reproductive Medicine Center of Fujian Maternity and Child Health Hospital from January 2018 to January 2022. Among them were 699 cases of clinical pregnancy and 931 cases of non-clinical pregnancy. The individuals were randomly sorted into a model group (n = 1141) and a validation group (n = 489) in a ratio of 7:3.

Inclusion Criteria: Infertile women diagnosed with endometriosis based on laparoscopy. endometriosis patients were divided into stages I-IV per the revised American Society for Reproductive Medicine (rAFS) staging system;patients who received IVF and fresh embryo transfer; those with complete data.

**Fig. 1:**
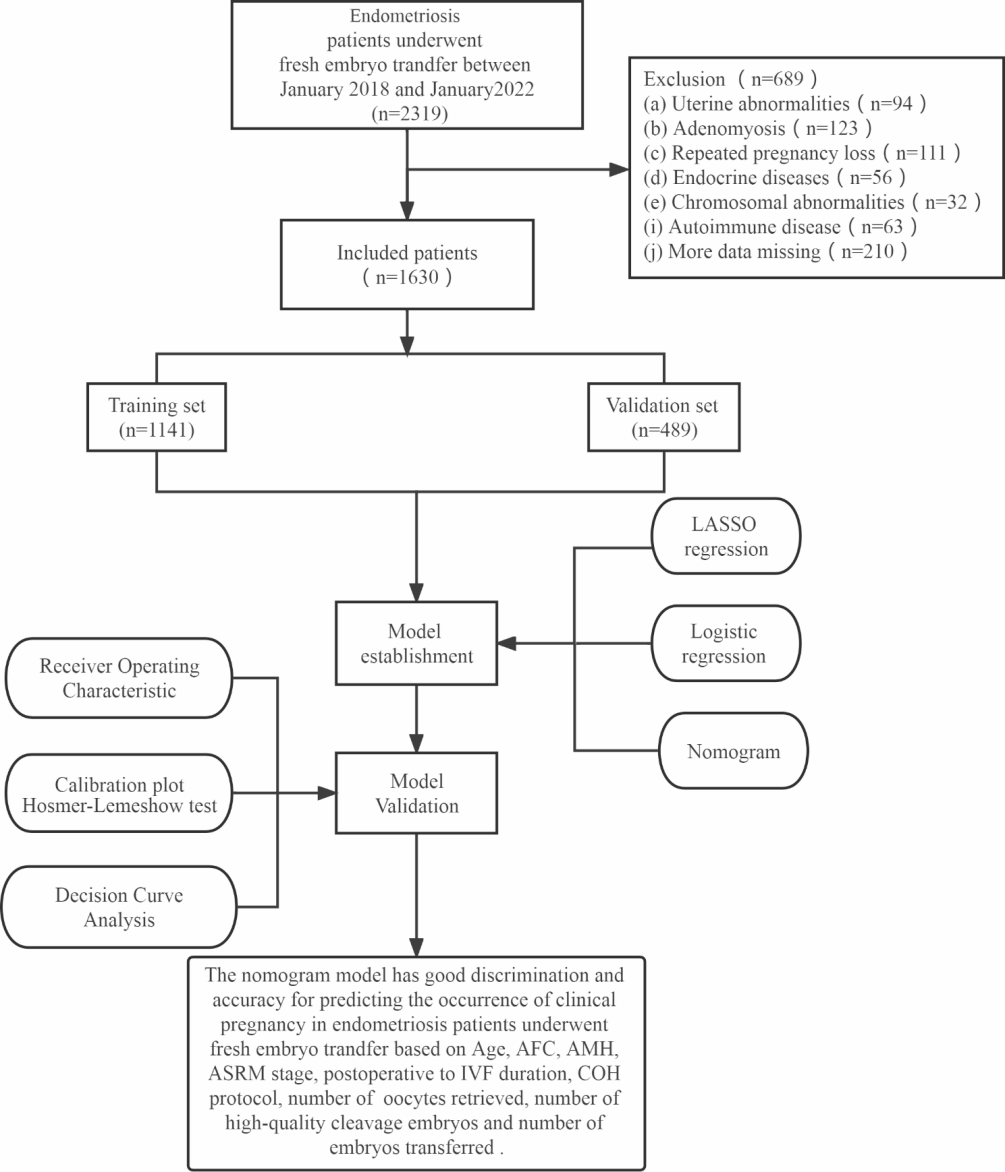
Research pathway diagram

Exclusion Criteria: Uterine fibroids ≥ 4 cm; untreated endometrial lesions; severe adenomyosis; uterine malformations; endocrine metabolic disorders; chromosomal abnormalities; autoimmune diseases; recurrent miscarriage.The research pathway is illustrated in Fig. 1.

### Methods

#### Controlled ovarian stimulation

Long-acting agonist group: On the second or third day of the menstrual cycle, a 3.75 mg long-acting Gonadotrophin-releasing hormone (GnRH) agonist (Triptorelin Acetate for Injection, Dophereline, IPSEN Pharmaceuticals, France) was administered. A follow-up visit was scheduled 30 days after medication administration to confirm the attainment of pituitary downregulation criteria (serum E2 < 50 pg/mL, LH < 5 U/L, endometrial thickness < 5 mm). Based on the ovarian reserve status of the patients and their previous response to controlled ovarian stimulation (COS), exogenous gonadotropins (Gn) were administered at a dose of 150–300 U. The selection encompassed Recombinant Follitropin Beta Injection (Puregon, Merck Serono, Germany), Recombinant Human Follitropin for Injection (Gonal-f, Merck Serono, Switzerland), or Urofollitropin for Injection (Lishenbao, Livzon Pharmaceutical Group, China). The dosage was adjusted per the ovarian response observed after five days of stimulation. Ovarian follicle growth monitoring involved serum hormone level measurements and transvaginal ultrasound examinations. When two ovarian follicles attained a diameter of ≥ 18 mm, or when three ovarian follicles achieved a diameter of ≥ 17 mm, a 250 μg recombinant HCG injection (Recombinant Human Choriogonadotropin for Injection, Azer, Merck Serono, Switzerland) was administered, and oocyte retrieval was conducted 36 h later.

Antagonist group: Exogenous Gn (150–300 IU) was administered starting from the 2nd or 3rd day of the menstrual cycle. On stimulation day 5, a 0.25 mg antagonist (Cetrorelix acetate for injection, Cetrotide, Merck Serono, Switzerland; or Ganirelix Acetate Injection, Ganirelix, Merck Serono, Germany) was added and continued until the trigger day. The initial dose of exogenous Gn was 150–225 IU, with subsequent adjustments based on follicle growth monitoring. The monitoring and ovarian follicle growth and the use of human chorionic gonadotropin (HCG) were similar to the long-acting agonist group.

#### Oocyte retrieval, IVF/ICSI fertilization, and embryo culture

Oocyte retrieval was conducted 36–37 h following the administration of the HCG injection, with the procedure guided by transvaginal ultrasound. The IVF artificial insemination was performed in the embryology laboratory following the standard operating procedures (SOP) of our center. The cleavage stage embryo grading criteria were primarily based on morphological indicators such as the number of blastomeres, blastomere uniformity, degree of fragmentation, and presence of multinucleation [6]. The embryos were divided into 4 grades: (1) Grade I embryos: 6 ~ 9 blastomes, uniform size, fragmentation degree 0 ~ 5%; (2) Grade II embryos: the size is basically uniform, and the degree of fragmentation is 10–25%; (3) Grade III embryos: uneven blastomere, fragmentation degree of 25% ~ 50%; (4) Grade IV embryos: blastomere is very uneven, fragmentation degree > 50%. Grade I and II embryos classified as high-quality embryos. The observation indexes included the size of blastocyst cavity, inner cell mass and trophoblast cells [7]. The blastocysts rated as 4, 5, 6(AA, AB, BA, BB) belong to the high quality blastocysts.

#### Identification of pregnancy outcome

A transvaginal ultrasound examination was performed 28 days following embryo transfer, and the presence of a gestational sac within the uterine cavity was defined as a clinical pregnancy.

#### Main outcome measures

The reproductive medical record management system of the hospital was used to collect clinical and laboratory data from both male and female partners from January 2018 to January 2022. The main variables included male age, female age, body mass index (BMI), ASRM stage, type of infertility, postoperative to IVF duration, stimulation protocol, AMH, AFC, basal luteinizing hormone, basal estradiol, basal follicle-stimulating hormone, starting dose of gonadotropins, total dose and duration of gonadotropin use, luteinizing hormone on the day of HCG injection, follicle-stimulating hormone on the day of HCG injection, estradiol on the day of HCG injection, progesterone on the day of HCG injection, endometrial thickness on the day of HCG injection, fertilization method, number of oocytes retrieved, stage of embryos transferred, number of transferred embryos and number of high-quality cleavage embryos.

#### Statistical analysis

Statistical analysis was conducted utilizing R 4.1, SPSS 26.0, GraphPad 8.0 and Stata 15.0 software packages for data processing and graphical representation. Firstly, the Kolmogorov-Smirnov test and Levene’s test were utilized to analyze the normality and homogeneity of variances in the data. Non-normally distributed continuous variables were presented as M (P25, P75), while categorical variables were expressed as n/%. Factors influencing clinical pregnancy in patients with endometriosis were screened using univariate analysis. Lasso regression with ten-fold cross-validation and lambda 1se as the criterion was employed to select the optimal combination of influencing factors. Subsequently, multiple forward stepwise logistic regression was then conducted to further examine the selected factors from Lasso regression. This analysis aimed to identify the independent factors that impact clinical pregnancy in individuals with endometriosis undergoing IVF/ICSI fresh transfer cycles.

Additionally, a nomogram for the model group was established. The sample was randomly split into model and validation groups in a 7:3 ratio. The area under the receiver operating characteristic (ROC) curve (AUC) assessed the discrimination of the model group and validation group. The Hosmer-Lemeshow test was utilized to examine the statistical difference between predicted and actual probabilities. The clinical efficacy was evaluated using clinical decision curves and clinical impact curves. *P <* 0.05 was deemed as a statistically significant value.

## Results

### Univariate analysis of clinical pregnancy in endometriosis patients undergoing fresh embryo transfer

The univariate analysis of factors influencing clinical pregnancy in patients with endometriosis demonstrated significant associations ( *P* < 0.05) with the following variables: female age, male age, ASRM stage, postoperative to IVF duration, stimulation protocol, AMH, AFC, basal follicle-stimulating hormone, starting dose of gonadotropins, duration of gonadotropin use, follicle-stimulating hormone on the day of HCG injection, luteinizing hormone on the day of HCG injection, estradiol on the day of HCG injection, endometrial thickness on the day of HCG injection, number of oocytes retrieved, number of high-quality cleavage embryos and number of transferred embryos (a total of 17 factors). The results are presented in Table 1.

**Table 1:** Univariate analysis of influencing factors of Clinical pregnancy

Characteristics	Total (n = 1630)	Unpregnancy (n = 931)	Pregnancy (n = 699)	P value
Female age(year)	33.0 [30.0;36.0]	34.0 [31.0;38.0]	32.0 [30.0;35.0]	<0.001*
Male age(year)	35.0 [31.0;38.0]	35.0 [32.0;40.0]	34.0 [31.0;37.0]	<0.001*
BMI(kg/m2), % (n)				0.375
>24	82.1 (1339)	81.4 (758)	81.3 (581)	
≤24	19.7 (291)	18.6 (173)	16.9 (118)	
Type of infertility, % (n)				0.203
Primary infertility	48.9 (797)	47.5 (442)	50.8 (355)	
Secondary infertility	51.1 (833)	52.5 (489)	49.2 (344)	
ASRM stage, % (n)				<0.001*
I/II	24.3 (396)	17.8 (166)	32.9 (230)	
III/IV	75.7 (1234)	82.2 (765)	67.1 (469)	
Postoperative to IVF duration(year), % (n)				<0.001*
≤3	72.0 (1173)	63.1 (587)	83.8 (586)	
>3	28.0 (457)	36.9 (344)	16.2 (113)	
COH protocol, % (n)				<0.001*
GnRH-antagonist	38.4 (568)	42.5(396)	24.6 (172)	
GnRH-agonist	65.2 (1062)	57.5 (535)	75.4 (527)	
Initial dose of Gn	200 [150;225]	225 [150;225]	188 [150;225]	<0.001*
Total dose of Gn	2700 [2175;3372]	2688 [2175;3300]	2700 [2144;3431]	0.283
Dosing days of Gn	12.0 [10.0;13.0]	11.0 [10.0;13.0]	12.0 [10.0;13.0]	<0.001*
Basal FSH (IU/L)	6.81 [5.73;8.29]	6.92 [5.79;8.43]	6.62 [5.66;8.02]	0.005 *
Basal LH (IU/L)	3.10 [2.30;4.20]	3.10 [2.30;4.20]	3.10 [2.30;4.20]	0.958
Basal E2 (pg/mL)	34.0 [25.0;47.0]	34.0 [25.0;47.0]	33.0 [24.0;46.0]	0.429
AFC	6.00 [4.00;7.00]	5.00 [3.00;7.00]	6.00 [5.00;8.00]	<0.001 *
AMH (μg/L)	1.71 [1.06;2.65]	1.54 [0.91;2.37]	2.02 [1.26;3.08]	<0.001*
FSH on HCG day (IU/L)	14.6 [11.7;18.0]	14.9 [12.1;18.5]	14.2 [11.1;17.3]	<0.001 *
E2 on HCG day (pg/mL)	1328 [773;1944]	1211 [724;1828]	1479 [861;2106]	<0.001*
LH on HCG day (IU/L)	1.20 [0.90;2.00]	1.30 [0.90;2.20]	1.10 [0.80;1.80]	<0.001*
P on HCG day (ng/mL)	0.47 [0.30;0.70]	0.46 [0.30;0.70]	0.49 [0.30;0.66]	0.849
Endometrial thickness on HCG day(mm)	11.0 [9.70;12.2]	11.0 [9.50;12.0]	11.0 [10.0;12.7]	<0.001 *
Fertilization, % (n)				0.113
IVF	78.5 (1279)	77.0(717)	80.4 (562)	
ICSI	21.5 (351)	23.0 (214)	19.6 (137)	
Number of oocytes retrieved	5.00 [4.00;7.00]	5.00 [3.00;7.00]	6.00 [4.00;7.00]	<0.001*
Number of high-quality cleavage embryos	3.00 [2.00;5.00]	3.00 [2.00;4.00]	4.00 [3.00;5.00]	<0.001 *
High-quality embryo rate, % (n/N)	81.4 (5843/7178)	80.2 (2978/3715)	82.7 (2865/3463)	0.371
Stage of embryos transferred, % (n)				0.088
Blastocyst	18.8 (307)	17.4 (162)	20.7 (145)	
Cleavage	81.2 (1323)	82.6 (769)	79.3 (554)	
Number of embryos transferred, % (n)				<0.001*
1	34.8 (567)	43.5 (405)	23.2 (162)	
2	65.2 (1063)	56.5 (526)	76.8 (537)	

^*^P < 0.05

**Fig. 2:**
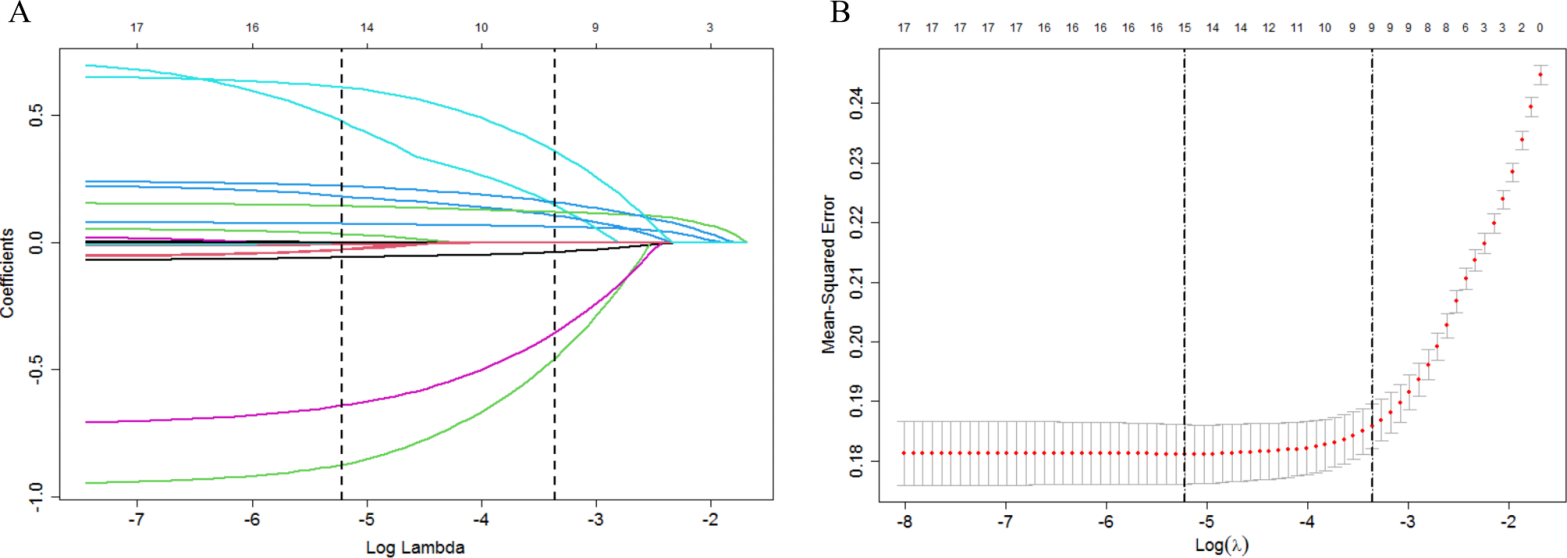
Best match factor screening by lasso regression. **A** is the Lasso regression path diagram; **B** shows the plot of the best matching factors screened by the ten-fold cross validation method, and the best matching factors were selected using lambda.1se as the criterion

Lasso regression was employed to further analyze the factors mentioned above. The model identified the following as the best matching factors: female age, ASRM stage, postoperative to IVF duration, stimulation protocol, AFC, AMH, E2 on HCG day, number of oocytes retrieved, number of high-quality Cleavage embryos and number of transferred embryos (a total of 9 factors), as shown in Fig. 2.

### Multivariable analysis of clinical pregnancy in endometriosis patients undergoing fresh embryo transfer

**Fig. 3:**
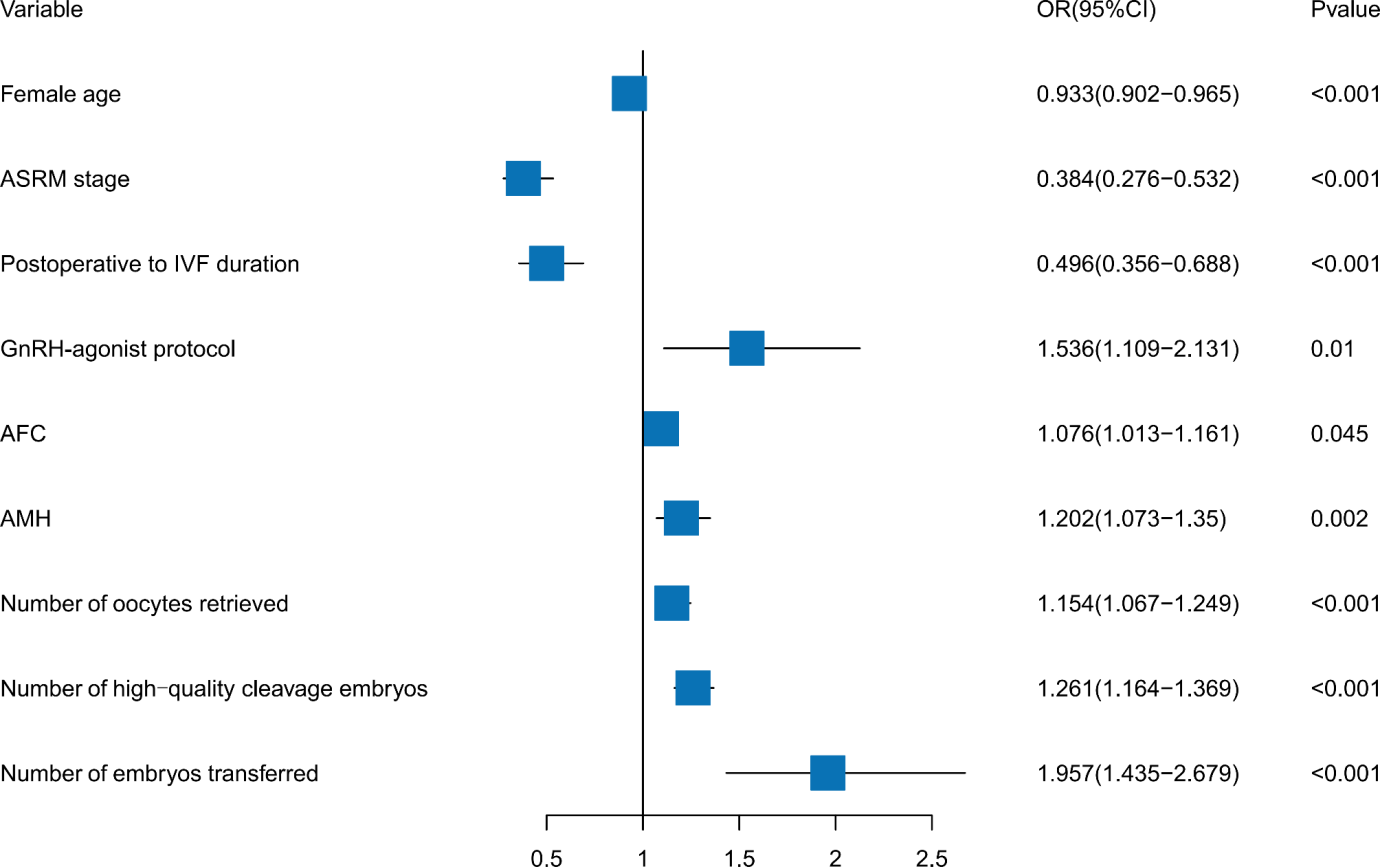
Forest plots of independent influencing factors for Clinical pregnancy by multivariate analysis Univariate

Through multivariable logistic regression analysis, female age (OR = 0.933, 95% CI = 0.902–0.965, *P* < 0.001), ASRM stage (OR = 0.384, 95% CI = 0.276–0.532, *P* < 0.001), postoperative to IVF duration (OR = 0.496, 95% CI = 0.356–0.688, *P* < 0.001), AFC (OR = 1.076, 95% CI = 1.013–1.161, *P* = 0.045), AMH (OR = 1.202, 95% CI = 1.073–1.35, *P* = 0.002), GnRH-agonist protocol (OR = 1.536, 95% CI = 1.109–2.131, *P* = 0.01), number of oocytes retrieved (OR = 1.154, 95% CI = 1.067–1.249, *P* < 0.001), number of high-quality cleavage embryos (OR = 1.261, 95% CI = 1.164–1.369, *P* < 0.001) and number of embryos transferred (OR = 1.957, 95% CI = 1.435–2.679, *P* < 0.001) were determined as independent factors influencing the clinical pregnancy rate in individuals with endometriosis (Fig. 3). Among these factors, the GnRH-agonist protocol, AFC, AMH, number of oocytes retrieved, number of high-quality cleavage embryos and number of embryos transferred were determined as independent protective factors for clinical pregnancy in endometriosis patients. Conversely, female age, ASRM stage, and the time interval between surgery and IVF were identified as independent risk factors (Fig. 3).

### Developing a nomogram model for predicting clinical pregnancy in endometriosis patients undergoing fresh embryo transfer

**Fig. 4:**
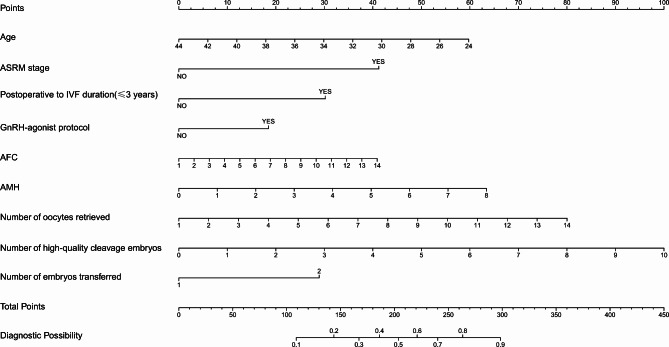
Nomogram of the prediction model for clinical pregnancy in endometriosis patients undergoing fresh embryo transfer

As per the findings of multivariate logistic regression, a nomogram was developed to predict the clinical pregnancy outcome in endometriosis patients. In personalized healthcare, the specific values of female age, ASRM stage, postoperative to IVF duration, stimulation protocol, AFC, AMH, number of oocytes retrieved, number of high-quality cleavage embryos and number of embryos transferred can be used to determine the corresponding points on the nomogram. By summing up the points from each variable, the nomogram estimates the clinical pregnancy probability for endometriosis patients undergoing fresh transfer cycles, as shown in Fig. 4.

### Validation of accuracy and discrimination of nomogram model

**Fig. 5:**
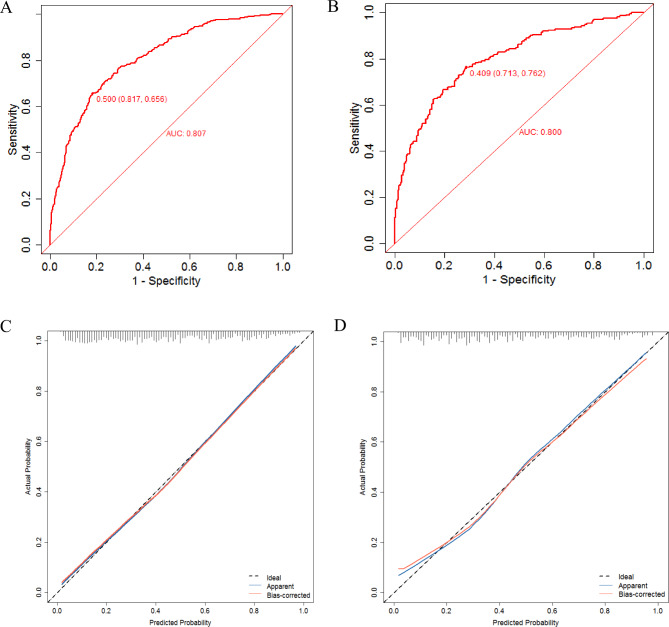
Discriminative power and accuracy of nomogram of the prediction model for clinical pregnancy in endometriosis patients undergoing fresh embryo transfer. **A** and **B** show the receiver operating curve of the model group and the validation group, respectively; **C** and **D** are calibration curves of the model group and the validation group, respectively

A random sampling was performed, with a ratio of 7:3 between the model group (n = 1141) and the validation group (n = 489). The accuracy and discrimination of the nomogram were validated using ROC and calibration curves. The results showed that AUC was 0.807 (95% CI = 0.782–0.832) for the model group and 0.800 (95% CI = 0.761–0.84) for the validation group, suggesting an enhanced discrimination ability of the prediction model. The Hosmer-Lemeshow test revealed no statistically significant difference between the predicted and observed clinical pregnancy probabilities in both the model group (R2 = 8.552, *P =* 0.479 > 0.05) and the validation group (R2 = 11.911, *P =* 0.218 > 0.05), suggesting that the model accurately predicted the clinical pregnancy probability in endometriosis patients (Fig. 5).

### Clinical efficacy evaluation of the clinical pregnancy nomogram model in endometriosis patients undergoing fresh embryo transfer

**Fig. 6:**
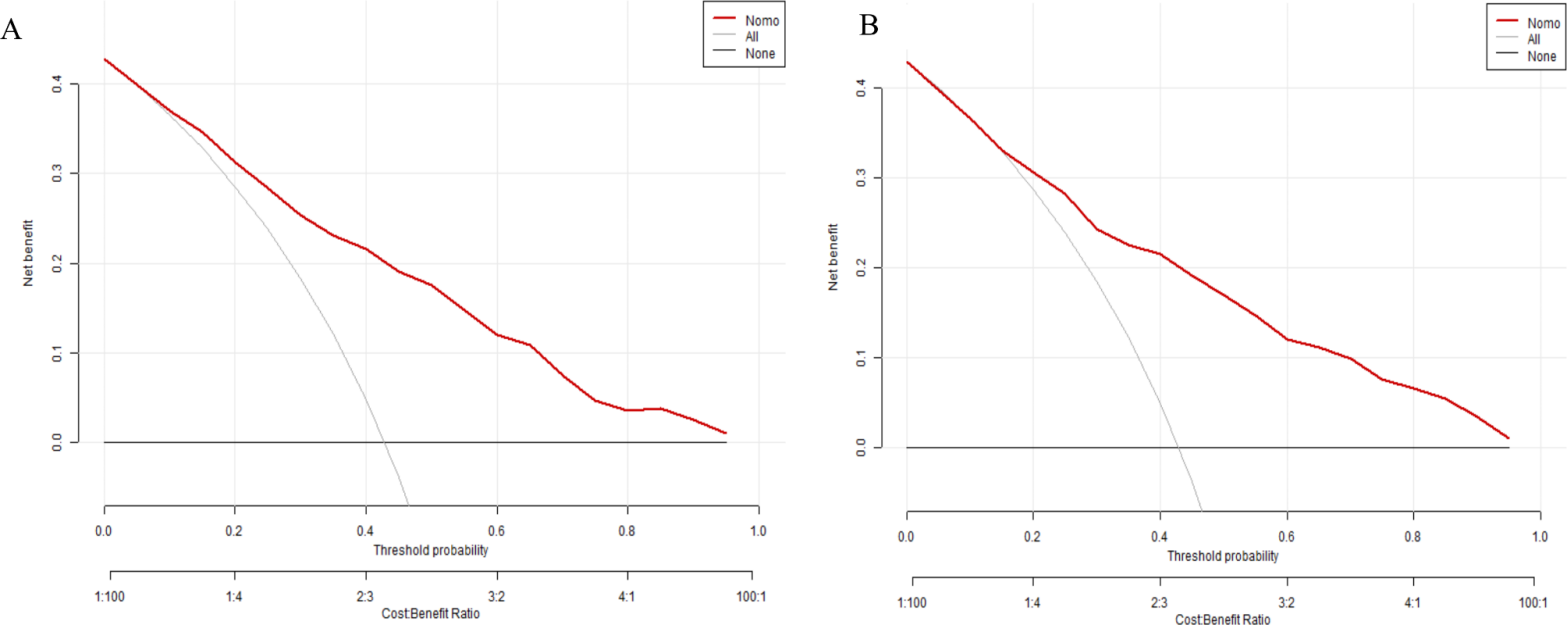
Discriminative power and accuracy of nomogram of the prediction model. **A** and **B** show the clinical decision curves of the model group and the validation group, respectively

The clinical efficacy of the model was analyzed using clinical decision curve analysis. The outcomes revealed that both the model and the validation groups achieved maximum benefits at threshold probability values ranging from 0.08 to 0.96 and 0.16 to 0.96. Within this threshold probability range, the number of predicted clinical pregnancies exceeded the number of actual high-quality blastocyst formations. Moreover, the loss-to-gain ratio remained consistently less than 1, indicating good clinical efficacy of the model (Fig. 6).

## Discussion

Endometriosis is a multifaceted clinical syndrome characterized by the involvement of multiple factors in its development. The specific etiology and underlying mechanisms of endometriosis remain unclear. Current evidence suggests that factors such as prenatal exposure to estradiol, short menstrual cycles, and smoking are high-risk factors for endometriosis. Additionally, low birth weight, early menarche, and smoking are also associated with an increased risk of Endometriosis development [8]. Many infertile patients are diagnosed with endometriosis when seeking infertility treatment. Moreover, infertility is an independent influencing factor for the diagnosis of mild endometriosis [9]. It is widely recognized that patients with endometriosis often experience poorer pregnancy outcomes [10, 11]. Predicting and enhancing ovarian responsiveness in these patients during ovulation induction has become an urgent concern for reproductive specialists. The aim is to attain improved pregnancy outcomes and address this crucial aspect of patient care.

This research conducted a multivariate logistic regression analysis to determine the independent influencing factors for the clinical pregnancy rate in fresh IVF/ICSI cycles of endometriosis patients. The factors that demonstrated a significant impact included female age, ASRM stage, postoperative to IVF duration, stimulation protocol, AFC, AMH, number of oocytes retrieved, number of high-quality cleavage embryos and number of embryos transferred. Per these independent factors, a nomogram model was constructed. This model quantifies and visualizes the outcomes of the logistic regression analysis and allows for the estimation of the values of independent variables, thereby predicting the probability of clinical pregnancy in individuals. The nomogram model is different from other clinical models in the past as it is more intuitive and practical. It enables clinicians to calculate the expected clinical pregnancy rate for the current treatment cycle based on the individual characteristics of the patient. This facilitates the adoption of personalized treatment plans to improve the clinical pregnancy rate. This approach can also reduce the psychological burden and economic pressure on patients [12].

The study revealed a considerable negative correlation between female age and pregnancy outcomes, indicating that older age is associated with poorer outcomes. Previous research has indicated that advanced female age can adversely affect oocyte quality and quantity, leading to an increased incidence of abnormal chromosome structure and number. Additionally, it can negatively impact endometrial receptivity. These factors collectively contribute to decreased clinical pregnancy and live birth rates in endometriosis patients [13]. A large-scale clinical study involving 51,959 fresh transfer cycles demonstrated that female age is the most significant factor contributing to decreased live birth rates and increased miscarriage rates [14]. In women aged over 35 years, there is a notable decline in ovarian response to gonadotropins, resulting in reduced oocyte quality and quantity. Furthermore, for women aged 40 and above, there is a significantly higher proportion of clinical low response, with rates reaching up to 50% [15]. Therefore, encouraging endometriosis patients to undergo assisted reproductive techniques at an earlier stage is crucial to enhance clinical pregnancy and live birth rates.

A cross-sectional study showed that AFC is significantly reduced in endometriosis patients compared to women without endometriosis [16]. AFC is one of the early predictive indicators used in ovarian stimulation cycles [17]. The POSEIDON criteria define AFC < 5 in both ovaries as diagnostic indicators of “expected poor ovarian response” and “unexpected poor ovarian response”. These criteria significantly affect the clinical pregnancy rate in individuals with poor ovarian response [18]. The current study also reflects the important role of AFC in predicting the clinical pregnancy rate in individuals with endometriosis. AMH, secreted by granulosa cells in growing pre-antral and small antral follicles, is currently considered the gold standard for assessing ovarian reserve, as it is not influenced by the menstrual cycle [19]. However, a retrospective cross-sectional study comparing ovarian responses in endometriosis patients and women with other causes of infertility undergoing IVF/ICSI stimulation showed that the ovarian response in endometriosis patients was considerably reduced than in the control group. This difference persisted even after adjusting for age, gonadotropin dosage, and AMH levels [20].

However, some research found that the AMH can only predict the number of oocytes but not their quality [21, 22]. In a study involving infertile patients undergoing IVF/ICSI, endometriosis significantly reduced AFC, AMH levels, retrieved oocytes, follicular maturation rate, and pregnancy rate compared to the control group. However, it did not affect the live birth rate. Moreover, preoperative removal of endometriosis before IVF/ICSI improved follicular maturation rate and pregnancy rate but did not increase the live birth rate [23]. In addition to AFC and AMH, basal FSH is another indicator of ovarian responsiveness in women. But in our study, basal FSH was not included in the model. Similar studies have previously concluded that FSH is to be inferior to AMH and AFC [24, 25]. Basal FSH levels are widely variable across menstrual cycles.

Severe endometriosis (classified as stage III-IV according to rAFS) was linked to a reduced cumulative clinical pregnancy rate per retrieval cycle [26]. This study also revealed a reduced clinical pregnancy rate in fresh transfer cycles for patients with rAFS stage III-IV, which may be attributed to the adverse effects of altered follicular fluid microenvironment on oocyte quality and developmental potential.Additionally, the severity of endometriosis may adversely affect the incidence of gestational diabetes, placenta previa, and small for gestational age in assisted reproductive technology (ART) pregnancies [27–29]. However, these conclusions are currently subject to debate as Geber et al. [30] found no correlation between IVF outcomes and the severity of endometriosis. Further detailed analysis and extensive research are needed in future studies.

There is still controversy regarding whether pre-implantation GnRH-a administration improves pregnancy outcomes in endometriosis patients. Studies have found that pre-treatment with GnRH-a before frozen embryo transfer in individuals with adenomyosis does not enhance clinical pregnancy and live birth rates [31]. Meta-analyses by Cao et al. [32] suggest that the GnRH-a long protocol in IVF-ET of endometriosis patients demonstrated improved clinical pregnancy rates in RCT studies. However, no notable variations were observed in non-RCT studies with different down regulation protocols. In the present research, the long agonist protocol was found to be an independent protective factor for clinical pregnancy in endometriosis patients, which may be related to the improvement of endometrial receptivity in endometriosis patients by the GnRH-a long protocol [33].

Furthermore, this study demonstrated a correlation between the time interval from laparoscopic surgery to IVF and pregnancy outcomes. The improved pelvic environment and enhanced endometrial receptivity in some endometriosis patients resulting from laparoscopic surgery may contribute to increased clinical pregnancy rates. However, as the time interval increases, the risk of endometriosis recurrence also increases. Additionally, young endometriosis patients who have obtained high-quality embryos can still achieve favorable clinical pregnancy rates. By increasing the number of embryos transferred, the clinical pregnancy rate can be further improved, which provides confidence for young endometriosis patients to pursue assisted reproductive techniques to conceive. Therefore, incorporating these independent influencing factors into the nomogram model is expected to obtain a more accurate predictive ability to guide clinical applications.

This study successfully developed a predictive nomogram model for the fresh cycle pregnancy outcome in patients with endometriosis, considering diagnostic criteria, individual parameters, and relevant laboratory tests. The model demonstrated an AUC of 0.807 (95% CI = 0.782–0.832) in the model group and an AUC of 0.800 (95% CI = 0.761–0.84) in the validation group, indicating a high discriminative ability and good predictive accuracy and specificity. It provides a more comprehensive reference for the development of clinical guidelines and medical decision-making. However, retrospective studies may introduce certain selection biases and result in potential deviations. Autoimmune diseases and other systemic comorbidities are factors affecting pregnancy rates, the model primarily predicts clinical pregnancy probabilities in endometriosis patients devoid of these comorbidities, albeit based on frequently utilized clinical parameters. Furthermore, this model necessitates external validation before clinical implementation.

In conclusion, the predictive nomogram model constructed in this study can be employed to predict and analyze pregnancy outcomes in endometriosis patients. Compared to traditional logistic regression models, the nomogram model is more simple, intuitive, and practical and can provide higher value in clinical applications.

## Data Availability

The datasets used and/or analyzed during the current study are available from the corresponding authors on reasonable request.
